# Efficiency of CIN2+ Detection by Thyrotropin-Releasing Hormone (TRH) Site-Specific Methylation

**DOI:** 10.3390/v15091802

**Published:** 2023-08-24

**Authors:** Arkom Chaiwongkot, Supranee Buranapraditkun, Shina Oranratanaphan, Thanaporn Chuen-Im, Nakarin Kitkumthorn

**Affiliations:** 1 Department of Microbiology, Faculty of Medicine, Chulalongkorn University, Bangkok 10330, Thailand; arkom.cha@chula.ac.th; 2 Center of Excellence in Applied Medical Virology, Faculty of Medicine, Chulalongkorn University, Bangkok 10330, Thailand; 3 King Chulalongkorn Memorial Hospital, Bangkok 10330, Thailand; bsuprane2001@yahoo.com; 4 Division of Allergy and Clinical Immunology, Department of Medicine, Faculty of Medicine, Chulalongkorn University, Bangkok 10330, Thailand; 5 Center of Excellence in Vaccine Research and Development (Chula Vaccine Research Center-(Chula VRC)), Faculty of Medicine, Chulalongkorn University, Bangkok 10330, Thailand; 6 Department of Obstetrics and Gynecology, Faculty of Medicine, Chulalongkorn University, Bangkok 10330, Thailand; dr_shina@hotmail.com; 7 Department of Microbiology, Faculty of Science, Silpakorn University, Nakhon Pathom 73000, Thailand; chuenim_t@su.ac.th; 8 Department of Oral Biology, Faculty of Dentistry, Mahidol University, Bangkok 10400, Thailand

**Keywords:** methylation, thyrotropin-releasing hormone, cervical cancer, CIN2+

## Abstract

Cervical cancer screening typically involves a Pap smear combined with high-risk human papillomavirus (hr-HPV) detection. Women with hr-HPV positivity but normal cytology, as well as those with precancerous abnormal cytology, such as low-grade squamous intraepithelial lesions (LSIL) and high-grade SIL (HSIL), are referred for colposcopy and histology examination to identify abnormal lesions, such as cervical intraepithelial neoplasia (CIN) and cervical cancer. However, in order to enhance the accuracy of detection, bioinformatics analysis of a microarray database was performed, which identified cg01009664, a methylation marker of the thyrotropin-releasing hormone (TRH). Consequently, a real-time PCR assay was developed to distinguish CIN2+ (CIN2, CIN3, and cervical cancer) from CIN2- (CIN1 and normal cervical epithelia). The real-time PCR assay utilized specific primers targeting methylated cg01009664 sites, whereas an unmethylated reaction was used to check the DNA quality. A cut-off value for the methylated reaction of Ct < 33 was established, resulting in improved precision in identifying CIN2+. In the first cohort group, the assay demonstrated a sensitivity of 93.7% and a specificity of 98.6%. In the cytology samples identified as atypical squamous cells of undetermined significance (ASC-US) and LSIL, the sensitivity and specificity for detecting CIN2+ were 95.0% and 98.9%, respectively. However, when self-collected samples from women with confirmed histology were tested, the sensitivity for CIN2+ detection dropped to 49.15%, while maintaining a specificity of 100%. Notably, the use of clinician-collected samples increased the sensitivity of TRH methylation testing. TRH methylation analysis can effectively identify women who require referral for colposcopy examinations, aiding in the detection of CIN2+.

## 1. Introduction

Cervical cancer is the fourth most frequent cancer in women worldwide, with more than 600,000 new cases, and more than 300,000 deaths reported in 2020 [1]. In Thailand, the incidence of new cervical cancer cases has decreased dramatically from 23.4 new cases in 1990 to 14 per 100,000 in the female population in 2014, making it the third most common cancer in Thai women [2]. It takes a long time for cervical precancerous lesions to progress to cervical cancer; the estimated median period of the progression of cervical cells from high-grade lesions (CIN2+) to cervical cancer was 23.5 years [3]. Cervical cancer screening has been performed by detecting the morphology of cervical cells, known as the Pap smear [4]. According to the Royal Thai College of Obstetricians and Gynecologists (RTCOG) guidelines, cervical cytology by conventional Pap smear or liquid-based cytology was recommended and reported, according to the Bethesda reporting system, classified as negative, atypical cells of undetermined significance (ASC-US), low-grade squamous intraepithelial lesions (LSIL), atypical squamous cells, and cannot exclude HSIL (ASC-H), high-grade squamous intraepithelial lesions (HSIL) and squamous cell carcinoma/adenocarcinoma. After colposcopy and biopsy examination, histology was reported as negative for intraepithelial lesions or malignancy, LSIL (or cervical intraepithelial neoplasia (CIN1), and HSIL (or CIN2+) [5,6]. Cytology alone, hr-HPV testing alone, or co-testing for cervical cancer screening are recommended by the RTCOG guidelines from 2020 [7]. However, cytology had a lower sensitivity than hr-HPV testing, and approximately 30% detected in the cytology were false negatives [8,9].

High-risk human papillomavirus (hr-HPV) has been detected in more than 99.0% of cervical cancer cases worldwide [10], with HPV16 being the most prevalent (>50% of cases), and HPV18 appearing in nearly 20% of all cases [11]. In 2014, the U.S. FDA approved the use of hr-HPV testing for screening for cervical cancer in women aged 25 and older. In women with ASC-US cytology, the American Society for Colposcopy and Cervical Pathology (ASCCP) guidelines recommended further hr-HPV testing before referral for colposcopy or repeat cytology within one year. Women with negative hr-HPV results were not referred for colposcopy, with a five-year interval for repeat screening [7,12,13,14,15]. However, the majority of women infected with HPV have a spontaneous regression; only a minority of those get cancer, and malignant transformation takes years [16,17]. The FDA-approved HPV testing assay was used in conjunction with cytology, known as “co-testing”, and could increase the sensitivity for detecting abnormal cervical lesions [12].

Women who have cytology results as LSIL/HSIL, or ASC-US with positive hr-HPVs will be referred for a colposcopy, and a biopsy will be collected for histological examination. The key issue is that only a minority of women infected with hr-HPVs developed cervical cancer [18]. The viral clearance rate in women with normal or ASC-US cytology with hr-HPV positivity was approximately 50%, while the CIN2+ progression was only 12% in women infected with a hr-HPV, and 25% in those with persistent hr-HPV infections [19]. A CIN2+ diagnosed histology was found in around 5–35.6% of LSIL/ASC-US cytology examinations [20,21,22,23,24,25]. CIN2+ lesions were seen in approximately 23.9% and 25.4% of HPV+/ASC-US- and solely HPV16/18-positive individuals, respectively [23]. Almost half of the ASC-US women had normal histologically confirmed results [25], and the incidence of cervical cancer in women with an ASC-US cytology was as low as 0.0–0.2% [26,27]. However, in a region with a high prevalence of cervical cancer, the incidence of cervical cancer in an ASC-US cytology was 7.9% [22]. To minimize unnecessary colposcopy referrals, additional tests, in addition to hr-HPV tests were developed, such as p16ink4a immunostaining alone [28], or p16/ki67 dual staining [29,30], hr-HPV oncogene expressions [31,32], and DNA methylation [33,34].

In this study, we aimed to detect specific CpG methylation of cellular genes as a biomarker in cervical samples with histologically confirmed lesions, compared to hr-HPV testing, in order to increase the positive predictive values of CIN2+ detection. The comprehensive methylation analysis utilized the GSE dataset of cervical samples, and a specific CpG site was selected for further exploration as an additional biomarker.

## 2. Materials and Methods

### 2.1. Bioinformatics

We collected methylation microarray data using two platforms: GPL8490 (Illumina^®^ HumanMethylation27 Bead Chip Kit, Illumina Inc., San Diego, CA, USA) and GPL13534 (HumanMethylation450 Bead Chip Kit, Illumina Inc., San Diego, CA, USA). The data were related to cervical cancer, normal cervical epithelium, and HPV. The inclusion criteria for the study encompassed normal, precancerous, and cancerous epithelia of cervical cancer. Conversely, the exclusion criteria involved cell lines, stem cells, blood cells, non-human tissue, non-cervical tissue samples, and congenital diseases. By accessing GENBANK (http://www.ncbi.nlm.nih.gov/geo, accessed on 1 November 2022), we obtained five series of experiments (GSEs) with the following identifiers: GSE41384 [35], GSE36637 [36], GSE30760 [37,38,39], GSE37020 [39,40], and GSE211668 [41]. Subsequently, the GEO2R tool was employed for data analysis. Utilizing methylation profiling datasets that comprised over 27,000 CpG positions, the methylation average of each CpG in the cervical samples ranging from normal to cervical cancer was analyzed. All analyzed samples are shown in Table 1. Then, graphs correlating methylation levels and the cervical lesion severities were created. Ultimately, we observed that cg01009664, which corresponds to the thyrotropin-releasing hormone (TRH), distinctly distinguished the differences in methylation percentage values between normal cervical epithelial cells and cancerous cells.

### 2.2. Clinical Specimens

Entire cervical samples were collected from women attending the King Chulalongkorn Memorial Hospital (Department of Obstetrics and Gynecology, Faculty of Medicine, Chulalongkorn University, Bangkok, Thailand) during 2017–2019, including 797 samples where cytology was confirmed. All samples were from leftover Pap smear specimens. In cases where HPV was positive or there was abnormal cytology, the definite diagnosis was gained by histological results from a colposcopy-directed biopsy or excisional procedure. Histological results included normal cervical tissue, HPV-positive lesions, and low-grade cervical lesions, classified as CIN2-. High-grade cervical lesions, including CIN2, CIN3, and cervical cancer, were classified as CIN2+.

The study included four cohorts for sample retrieval and analysis. Cohort 1, referred to as the “discovery set”, consisted of routine Pap smear samples. The total number of samples in this cohort was 297, with 218 samples diagnosed as CIN2- and 79 samples diagnosed as CIN2+. Cohort 2 comprised samples that were cytologically diagnosed as ASC-US/LSIL. These samples were leftovers from a previous study [42]. The total number of samples in this cohort was 209, with 189 samples diagnosed as CIN2- and 20 samples diagnosed as CIN2+.

Cohort 3 and cohort 4 also consisted of leftover specimens from a previous study [43]. Cohort 3 comprised patient self-collected samples for a total of 275. Among these samples, 216 were diagnosed as CIN2- and 59 were diagnosed as CIN2+. Cohort 4 comprised samples collected by healthcare professionals, for a total of 126 samples. Among these samples, 92 were diagnosed as CIN2- and 34 were diagnosed as CIN2+. The study was conducted in accordance with the Declaration of Helsinki, and the protocol was approved by the Ethics Committee of the Institutional Review Board (IRB) of the Faculty of Medicine, Chulalongkorn University (COA No. 087/2016 and COA No. 389/2017).

### 2.3. hr-HPV Detection by Cobas^®^ 4800 HPV-DNA Assay

The hr-HPV DNA test was performed by Cobas^®^4800 HPV testing, a fully automated test based on the extraction of HPV and cellular DNA, followed by real-time PCR. The assay can detect HPV16 and HPV18, as well as the presence of a pool of twelve other hr-HPVs (HPV31, 33, 35, 39, 45, 51, 52, 56, 58, 59, 66, and 68). To confirm sample quality, the beta-globin gene was employed as a genomic DNA control. After that, the remaining DNA was submitted for bisulfite modification.

### 2.4. DNA Bisulfite Modification

The DNA concentration was assessed using a nanodrop and later modified to reach a concentration of 750 ng/µL. To carry out the bisulfite treatment, 20 µL of each sample was treated with the EZ DNA Methylation Kit (Zymo Research, Irvine, CA, USA). The resulting converted DNA solutions were eluted in 20 µL of M-Elution Buffer and stored at temperatures below −20 °C for future utilization.

### 2.5. Detection of TRH Methylation by Real-Time Polymerase Chain Reaction (PCR)

Real-time PCR in position cg01009664 was performed as previously described [44]. In brief, we employed real-time methylation-specific PCR using the Sensi-FastTM SYBR^®^ Low-Rox Kit (Bioline, Alexandria, NSW, Australia). PCR reactions were prepared in a 20 μL volume, comprising 10 μL of 2X Sensi-FastTM SYBR^®^ Low-Rox reagent, 0.8 μL of 500 nmoles of each gene-specific forward and reverse primers, and 1 μL of bisulfite-treated DNA template. The remaining volume was adjusted by adding milliQ DNase-free sterile water. Two sets of gene-specific primers were utilized: one set targeting the methylated state of the target gene (met forward primer: 5′-ATT CGG GGA TTC GGG ATT C-3′, met reverse primer: 5′-GAC GAC CCA TCT AAA AAA AAC TCG-3′), and the other set targeting the unmethylated state of the target gene (unmet forward primer: 5′-GGA TTT GGG GAT TTG GGA TTT-3′, unmet reverse primer: 5′-CAC TCA AAC CAC CAC CTA ACA-3′).

Real-time PCRs were conducted in duplicate using an Applied Biosystem^®^ 7500 Real-Time PCR System (Thermo Scientific™, Waltham, MA, USA). Each PCR run included a no-template control and a positive control to monitor for potential contamination. The PCR conditions consisted of 45 cycles, with denaturation at 95 °C for 2 min, followed by annealing at 60 °C for 30 s. Fluorescence signals from the amplified product were detected. A melting curve analysis was performed to ascertain primer specificity. The threshold cycle (Ct) was determined for the amplified methylation products. The methylation-specific reaction was measured by Ct value, whereas the unmethylation-specific reaction was used to check DNA quality and integrity.

### 2.6. Statistical Analysis

Statistical analysis was performed using GraphPad Prism 9 (GraphPad Software, Inc., San Diego, CA, USA). All information was analyzed as a mean and percentage. A receiver operating characteristic (ROC) curve was constructed to evaluate the diagnostic capability of gene methylation levels in distinguishing between CIN2+ and CIN2-. The diagnosis performance of the hr-HPV test and TRH methylation for the detection of CIN2+ was reported, with respect to sensitivity, specificity, positive predictive value (PPV), and negative predictive value (NPV) using a 95% confidence interval.

## 3. Results

### 3.1. TRH Methylation Cut-Off Determination

**Figure 1:**
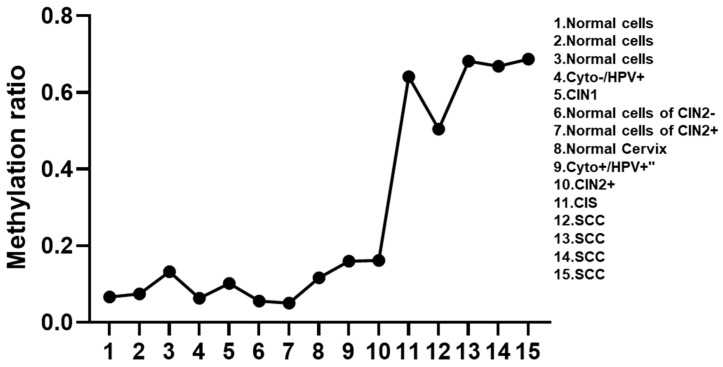
Methylation levels at cg01009664 of TRH in cervical cells with different cervical lesions. Denoted: CIN: cervical intraepithelial neoplasia; SCC: squamous cell carcinoma.

After analyzing the methylation ratio of more than 27,000 CpG positions in five GSE datasets, we searched for CpG positions with a remarkable ability to distinguish between CIN2- and CIN2+. Compared to normal cervical cells, the cg01009664 location of thyrotropin-releasing hormone (TRH) was significantly methylated in cervical cancer cases (Figure 1).

**Figure 2:**
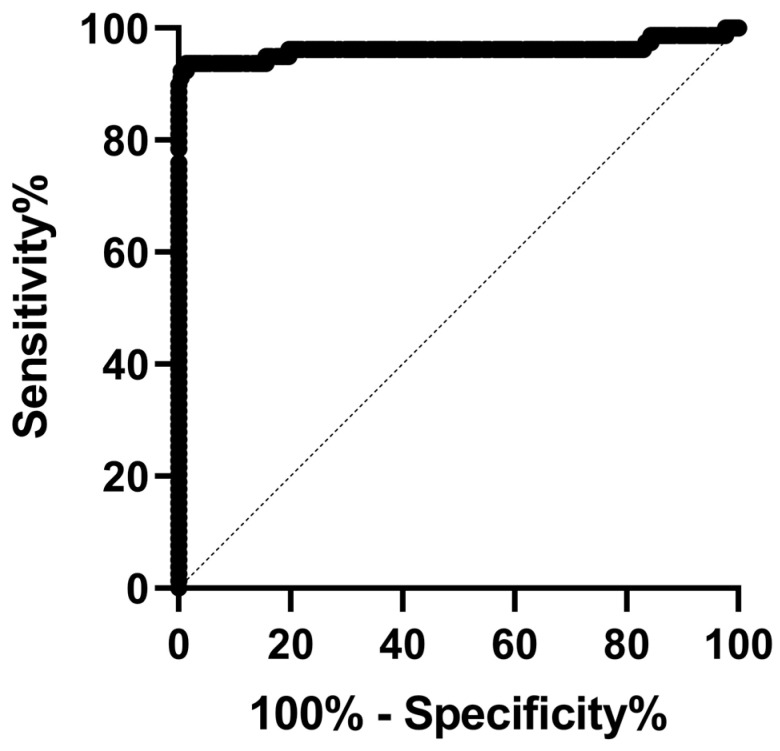
Sensitivity and specificity of TRH methylation patterns to differentiate between CIN2- and CIN2+ lesions. AUC was 0.961 (95% CI: 0.923–0.999) (*p*-value < 0.0001).

Next, real-time PCR was developed to optimize the conditions and define the cut-off TRH methylation Ct value. This feature has the potential to discriminate between CIN2- and CIN2+ lesions within the discovery set. Unmethylated TRH was identified in both the CIN2- and CIN2+ samples, whereas methylation of TRH was detected exclusively in high-grade lesion samples. The obtained Ct values from primers designed for detecting methylated TRH in samples categorized as CIN2- and CIN2+ were employed to construct ROC curves (see Table S1). These curves were analyzed to determine an appropriate cut-off value. From the TRH methylation reaction, the area under the ROC curve (AUC) was 0.962 (Figure 2). The best sensitivity and specificity values were 93.67% and 98.62%, respectively, with a Ct value of 33.0 (Table 2). Therefore, a Ct value of 33.0 or less was defined as CIN2+, while a Ct value greater than 33.0 was defined as CIN2-. Ultimately, we used this cut-off as a standard for the following experiments.

### 3.2. Diagnostic Performance of hr-HPV and TRH Methylation in Detecting for CIN2+ Lesions

To evaluate their utility in diagnosing CIN2+ lesions, hr-HPV detection, and TRH methylation were analyzed separately using cohort 1. The hr-HPV detection rates were 32.5% and 55.7% for CIN2- and CIN2+, respectively, whereas the TRH methylation rates were as low as 1.4% for CIN2- but had a high detection rate of 93.7% for CIN2+, in the initial batch of 297 samples used to optimize the cut-off point (Table 3). TRH methylation alone could improve the specificity and PPV in identifying CIN2+ lesions, as well as having a high sensitivity and NPV.

### 3.3. Detection of hr-HPVs and TRH Methylation for Diagnosing CIN2+ Lesions in the ASC-US/LSIL Group

Tests have been further applied to the ASC-US/LSIL cytology-diagnosed group (cohort 2) with histological-confirmed results. The hr-HPV detection rates for CIN2- and CIN2+ were high (82.2% and 90.0%, respectively) with low specificity and PPV (13.76% and 9.94%, respectively), whereas TRH methylation had a low detection rate for CIN2- and a high detection rate for CIN2+ (1.1% vs. 95.0%, respectively) with high sensitivity, specificity, PPV, and NPV. Combined testing (hr-HPV and TRH methylation) in this group could slightly improve specificity for identifying CIN2+ (Table 3 and Table 4).

### 3.4. Detection of hr-HPVs and TRH Methylation for Diagnosing CIN2+ Lesions in Self-Collected and Clinician-Collected Groups

In order to increase the test’s applicability for cervical cancer screening, self-collected specimens (cohort 3) were used to evaluate the clinical accuracy of TRH methylation, and the results were compared to those of clinician-collected specimens (cohort 4). TRH methylation and combination test rates were 0% for CIN2- and the detection rate for CIN2+ lesions was greater in clinician-collected samples than in self-collected samples (76.5% vs. 49.2%) (Table 3). Although TRH methylation and the combination tests had high specificity and PPV (100%), they had a modest sensitivity (38.98–49.15%) in the self-collected samples. The clinician-collected samples demonstrated higher sensitivity and NPV (Table 4). The hr-HPV detection rates were comparable in the self-collected and clinician-collected samples with CIN2- or CIN2+ (Table 3).

## 4. Discussion

Nowadays, cytology assays demonstrate limited sensitivity, whereas hr-HPV detection has low specificity. Using hr-HPV and cytology tests in combination did not improve the specificity of cervical cancer screening [45,46]. The CIN2+ detection rates in ASC-US and LSIL cytology groups with HPV-DNA positives were 14.2% and 12.0%, respectively, yet the HSIL cytology group had a high detection rate (67.8%) [47]. It would be interesting to develop a more precise biomarker to triage women with cytology diagnosed as ASC-US/LSIL and/or hr-HPV positive.

The present study used GSE datasets to scan for CpG methylation in cervical samples with varying degrees of lesion severity and discovered that methylation at the TRH cg01009664 site exhibited a greater difference between normal and cervical cancer patients. This position was previously reported to be hypermethylated in oral and oropharyngeal cancers and could be used to discriminate between oral cancer cells and normal cells detected by quantitative pyrosequencing and real-time PCR [44]. The comparable nature of this site could be due to the fact that both tumors are squamous epithelial in origin. As a result, this location may be critical in the transition from unmethylated to methylated in epithelial cells. However, the functional study must be further investigated. One study found that TRH was substantially methylated in pancreatic cancer cell lines by employing microarrays coupled with methyl-CpG targeted transcriptional activation (MeTA-array) and methylation-specific PCR [48]. Other investigations reported that TRH was also hypermethylated in clear cell renal cell carcinomas [49,50]. The present study revealed that TRH methylation had the highest sensitivity and specificity for detecting CIN2+ lesions compared to hr-HPVs alone. Other investigations, which used a panel of DNA methylation markers, such as CADM1, MAL, miR124, CCNA1, PAX1, SOC1, and HPVL1, also exhibited lower sensitivity than TRH methylation [33,34,42,43,51,52,53,54,55].

Other biomarkers for diagnosing CIN2+ have been explored, including the use of p16ink4a immunostaining on its own or in combination with p16/ki67 dual staining. In cases of LSIL women with hr-HPV-positivity, dual staining demonstrated a sensitivity and specificity range of 77.8–85.7% and 53.3–88.6%, respectively, for detecting CIN2+ lesions. For ASC-US women, the sensitivity and specificity for CIN2+ detection were found to be 71.9–94.4% and 78.7–87.9%, respectively [29,30,56]. Notably, in women outpatients, the sensitivity and specificity for CIN2+ detection were 90.2% and 68.3%, respectively [57]. Sole p16ink4a immunostaining displayed lower sensitivity (41%) and specificity (86%) in LSIL women compared to dual staining [28]. In addition, HPV16 E7 mRNA expression has also been associated with cervical lesion progression to CIN2+ and holds potential as a diagnostic marker for HSIL [31,32].

In this study, the diagnostic value of TRH testing alone demonstrated excellent performance across all four cohorts, especially in cohort 2 (ASC-US/LSIL), with high specificity ranging from 98% to 100%, high PPV ranging from 90% to 100%, and high NPV ranging from 87% to 99%. These results highlight the effectiveness of TRH testing as a diagnostic tool. However, it is important to note that the sensitivity of TRH testing was comparatively lower, ranging from 49% to 95%, particularly in self-collected samples. This finding aligns with previous research indicating that self-sampling for hr-HPVs and HSIL cytology tends to yield lower sensitivity compared to samples collected by healthcare professionals [58].

Despite the low sensitivity of TRH methylation in self-collected samples, its specificity and positive predictive value (PPV) are both 100%, indicating that a positive result for TRH methylation is highly indicative of a high-grade lesion in the patient. Specifically, in cases of ASC-US, TRH methylation testing demonstrates high percentages of sensitivity, specificity, NPV, and PPV, all of which are characteristics of a reliable diagnostic test. Therefore, when ASC-US is positive for TRH methylation, a further colposcopy is suggested for a definitive diagnosis.

In clinical practice, these positive results of TRH methylation could have various applications. One such application is the utilization of the TRH methylation test in cases of HPV DNA positivity, allowing for the classification of an urgent colposcopy in situations where there are long waiting times. In patients who test positive for TRH methylation, an urgent colposcopy may be necessary. Conversely, if results for both hr-HPV and TRH methylation are negative, a colposcopy may be omitted due to the high negative predictive value (NPV) of these tests. By implementing a screening test with high sensitivity and specificity, the utilization of a colposcopy in the diagnosis of an abnormal cervix in the low-risk group can be improved, leading to a reduction in the number of women referred for a colposcopy.

The human TRH is located on chromosome 3, TRH is produced in the hypothalamus and binds to its receptor at the anterior of the pituitary gland, activating G protein-coupled receptors (GPCRs) that stimulate the pituitary gland to release thyroid stimulating hormone (TSH). Then, TSH activates the thyroid gland, causing it to release the thyroid hormones thyroxine (T4) and triiodothyronine (T3), which affect homeostasis and energy metabolism and are necessary for appropriate growth and development. TRH also promotes prolactin and growth hormone secretion. T4 and T3 regulate TRH and TSH secretion via a negative feedback mechanism [59,60,61]. TRH was found to be downregulated in various cancers, including cervical squamous cell carcinoma, endocervical adenocarcinoma, and ovarian cancer, but abundantly expressed in leukemia [62]. According to one study, the thyrotropin-releasing hormone-degrading enzyme was shown to be expressed in malignant cervical cells but not in normal cervical cells [63]. TRH was utilized to treat cancer-related fatigue in patients and showed considerable improvement [64,65].

In this study, we found methylation at cg0100966 of TRH in high-grade cervical lesions. The study’s limitation is that we did not observe the TRH expression level in the samples. Therefore, the correlation between methylation and expression must be further validated. The underlying mechanism to clarify the importance of this methylation must also be studied in the future. As far as our knowledge extends, no investigation has been conducted to explore the involvement of TRH in cervical carcinogenesis from the perspectives of gene mutation, protein expression, and epigenetics. Our strength in this study is that a final pathological confirmation was performed in all abnormal cases, which was the gold standard in evaluating the diagnostic values. Moreover, we confirmed the diagnostic value of TRH in four cohorts. For future investigations, it would be beneficial to expand the cohort by including a larger number of patients diagnosed with CIN2+ in order to enhance the scope of the evaluation. Furthermore, exploring this subject with diverse population groups or across various geographic locations could provide valuable insights and a more comprehensive understanding. Consequently, this approach could potentially lead to more precise insights into diagnostic values and the potential role of triage.

In conclusion, TRH methylation testing in four cohort groups demonstrated the greatest sensitivity and specificity for identifying CIN2+ cervical lesions and could be utilized as an adjunct test to hr-HPV testing, particularly in ASC-US/LSIL women and prior to colposcopy referral.

## Figures and Tables

**Table 1:** Samples used for bioinformatics classified by cervical lesions.

Column	GSE *	Detail from Corresponding GSE	No.
1	41384	Normal cervical sample	3
2	36637	Genomic DNA from frozen normal ectocervix	4
3	30760	Cells from a normal uterine cervix	15
4	37020	HPV +ve/cyto −ve liquid-based cytology cervical smear samples	24
5	41384	Cervical intraepithelial neoplasia (CIN)1	3
6	30760	Normal cells of the uterine cervix CIN2-	75
7	30760	Normal cells of the uterine cervix CIN2+	77
8	41384	Cervical intraepithelial neoplasia (CIN)2	4
9	211668	Normal cervix	18
10	37020	HPV +ve/cyto +ve liquid-based cytology cervical smear samples	24
11	41384	Cervical carcinoma in situ	6
12	211668	Cervical cancer	63
13	41384	Invasive cervical carcinoma	3
14	36637	Genomic DNA from frozen squamous carcinoma	5
15	30760	Cells from a uterine cervix cancer	48

* GSE41384: methylation profiling in intraepithelial neoplasia and cervical cancer [1]; GSE36637: aberrant promoter methylation and expression of UTF1 during cervix carcinogenesis [2]; GSE30760: cervical cancer [3,4,5]; GSE37020: epigenome analysis of smear cells from the uterine cervix consisting of 24 normal and 24 cervical intraepithelial neoplasia (all HPV+) [4,6]; GSE211668: epigenome analysis of primary cervical cancer and normal [7].

**Table 2:** Sensitivity and specificity of TRH methylation cut-off points to differentiate between CIN2- and CIN2+ lesions.

Positive If Ct Is Lower than or Equal to	Sensitivity (%)	Specificity (%)	Positive If Ct is Lower than or Equal to	Sensitivity (%)	Specificity (%)	Positive If Ct Is Lower than or Equal to	Sensitivity (%)	Specificity (%)
32.10	81.01	100.00	34.48	93.67	94.50	36.51	94.94	84.40
32.17	82.28	100.00	34.53	93.67	94.04	36.69	94.94	83.94
32.22	83.54	100.00	34.60	93.67	93.58	36.79	94.94	83.49
32.23	84.81	100.00	34.67	93.67	93.12	36.87	94.94	83.03
32.27	86.08	100.00	34.72	93.67	92.66	36.96	94.94	82.57
32.35	87.34	100.00	34.75	93.67	92.20	37.08	94.94	82.11
32.43	88.61	100.00	34.86	93.67	91.74	37.19	94.94	81.65
32.48	89.87	100.00	34.96	93.67	91.28	37.23	94.94	81.19
32.57	89.87	99.54	35.20	93.67	90.83	37.28	94.94	80.73
32.72	91.14	99.54	35.51	93.67	90.37	37.38	94.94	80.28
32.82	92.41	99.54	35.62	93.67	89.91	37.47	96.20	80.28
32.88	92.41	99.08	35.71	93.67	89.45	37.51	96.20	79.82
32.93	92.41	98.62	35.82	93.67	88.99	37.54	96.20	79.36
33.07	93.67	98.62	35.94	93.67	88.53	37.57	96.20	78.90
33.23	93.67	98.17	36.07	93.67	88.07	37.59	96.20	78.44
33.40	93.67	97.71	36.15	93.67	87.61	37.63	96.20	77.98
33.56	93.67	97.25	36.18	93.67	86.70	37.69	96.20	77.52
33.61	93.67	96.79	36.21	93.67	86.24	37.76	96.20	77.06
33.65	93.67	96.33	36.24	93.67	85.78	37.88	96.20	76.61
33.68	93.67	95.87	36.26	93.67	85.32	37.99	96.20	76.15
33.92	93.67	95.41	36.27	93.67	84.86	38.11	96.20	75.69
34.30	93.67	94.95	36.33	93.67	84.40	38.23	96.20	75.23

**Table 3:** Percentage of TRH methylation and HR-HPV detection in CIN2- and CIN2+ lesions.

	CIN2-				CIN2+	
	hr-HPVs	TRH Met	hr-HPV /TRH Met	hr-HPVs	TRH Met	hr-Hpv /TRH Met
Cohort 1: discovering	32.5%	1.4%	0.9%	55.7%	93.7%	53.2%
	(71/218)	(3/218)	(2/218)	(44/79)	(74/79)	(42/79)
Cohort 2: ASC-US/LSIL	82.2%	1.1%	0.5%	90.0%	95.0%	90.0%
	(163/189)	(2/189)	(1/189)	(18/20)	(19/20)	(18/20)
Cohort 3: self-collected	61.1%	0.0%	0.0%	74.6%	49.2%	38.9%
	(132/216)	(0/216)	(0/216)	(44/59)	(29/59)	(23/59)
Cohort 4: clinician-collected	57.6%	0.0%	0.0%	76.5%	76.5%	64.7%
	(53/92)	(0/92)	(0/92)	(26/34)	(26/34)	(22/34)

**Table 4:** Diagnostic capacity for CIN2+ detection of TRH and/or hr-HPV detection.

	hr-HPVs				TRH Met				TRH Met and hr-HPVs			
	Sen (%)	Spec (%)	PPV (%)	NPV (%)	Sen (%)	Spec (%)	PPV (%)	NPV (%)	Sen (%)	Spec (%)	PPV (%)	NPV (%)
Cohort 1: discovering	55.70	67.43	38.26	80.77	93.67	98.62	96.10.	97.73	53.16	99.08	95.45	85.38
Cohort 2: ASC-US/LSIL	90.00	13.76	9.94	92.86	95.00	98.94	90.48	99.47	90.00	99.47	94.74	98.95
Cohort 3: self-collected	74.58	38.89	25.00	84.85	49.15	100.00	100.00	87.80	38.98	100.00	100.00	85.71
Cohort 4: clinician-collected	76.47	42.39	32.91	82.98	77.78	100.00	100.00	92.00	64.71	100.00	100.00	88.46

Denoted: Sen: sensitivity; Spec: specificity; PPV: positive predictive value; NPV: negative predictive value; Met: methylation; ASC-US: atypical squamous cells of undetermined significance; LSIL: low-grade squamous intraepithelial lesions.

## Data Availability

The data presented in this study are available upon reasonable request to the corresponding author.

## Supplementary Materials

The following supporting information can be downloaded at: https://www.mdpi.com/article/10.3390/v15091802/s1, Table S1. Supplement raw data.
